# Improvement of sleep in patients with chronic idiopathic/spontaneous urticaria treated with omalizumab: results of three randomized, double-blind, placebo-controlled studies

**DOI:** 10.1186/s13601-016-0120-0

**Published:** 2016-08-18

**Authors:** Ana M. Gimenéz-Arnau, Sheldon Spector, Evgeniya Antonova, Benjamin Trzaskoma, Karin Rosén, Theodore A. Omachi, Donald Stull, Maria-Magdalena Balp, Thomas Murphy

**Affiliations:** 1 Hospital del Mar, Institut Mar d’Investigacions Mèdiques, Universitat Autonoma Barcelona, Barcelona, Spain; 2 California Allergy and Asthma Medical Group, Inc., 11645 Wilshire Blvd, #1155, Los Angeles, CA 90025 USA; 3 Medical Affairs, Genentech, Inc., 1 DNA Way, South San Francisco, CA 94080-4990 USA; 4 Data Analytics and Design Strategy, RTI Health Solutions, Manchester, UK; 5 HEOR in Global Patient Access, Novartis Pharma AG, 4002 Basel, Switzerland; 6 National Allergy, Asthma, and Urticaria Centers of Charleston, 7555 Northside Drive, Charleston, SC 29420 USA

**Keywords:** Chronic idiopathic urticaria, Chronic spontaneous urticaria, Omalizumab, Sleep, Sleep quality

## Abstract

**Background:**

Patients with chronic idiopathic/spontaneous urticaria (CIU/CSU) report difficulty with sleep.

**Methods:**

We examined the effect of omalizumab on sleep-related outcomes during 3–6 months omalizumab or placebo treatment and a 16-week follow-up period within three Phase III double-blind randomized placebo-controlled pivotal trials in CIU/CSU: ASTERIA I, ASTERIA II, and GLACIAL. Sleep quality was assessed in all three studies using sleep-related questions included in an electronic diary, the Chronic Urticaria Quality of Life Questionnaire, and the Medical Outcomes Study Sleep Scale. Score changes from baseline in the treatment arms were compared with that in the placebo arm and adjusted for baseline score and weight. We also examined correlations of sleep scores at baseline, week 12, and week 24 and the slopes of change between sleep and itch and hive.

**Results:**

Patients treated with omalizumab reported a larger reduction in sleep problems than those in the placebo arm; omalizumab 300 mg demonstrated the greatest improvement on all sleep components among all treatment arms. The largest reduction in sleep problems was reported within the first 4 weeks of therapy. After treatment discontinuation, sleep quality worsened. Sleep scores demonstrated moderate-to-strong correlation between them, and the change in sleep scores was associated with changes in itch and hives.

**Conclusions:**

Improvement in sleep was reported after the first dose of omalizumab. Sleep continued to improve throughout the active treatment period. Patients receiving omalizumab 300 mg achieved greater improvement in sleep than those in other treatment arms.

*Trial registration* ClinicalTrials.gov, NCT01287117 (ASTERIA I), NCT01292473 (ASTERIA II), and NCT01264939 (GLACIAL)

**Electronic supplementary material:**

The online version of this article (doi:10.1186/s13601-016-0120-0) contains supplementary material, which is available to authorized users.

## Background

The Institute of Medicine has warned that chronic sleep disorder and wakefulness adversely impact health and longevity [1]. Many chronic conditions are associated with impairments of sleep quantity or quality [2–5]. Chronic idiopathic urticaria (CIU), also known as chronic spontaneous urticaria (CSU), is a disorder characterized by chronic hives, itch, and often angioedema that may lead to difficulty initiating or maintaining sleep or poor sleep quality [6–12]. CIU/CSU patients reported that the presence of hives, itching, or both prevented adequate sleep [7–11]. In these patients, poor sleep causes fatigue and diminishes physical and emotional well-being [6]. In an Internet survey of patients with chronic urticaria, 48 % of respondents reported that sleep disturbances related to chronic urticaria were not adequately addressed [9]. When compared with patients with heart disease, patients with chronic urticaria report more difficulty with sleep disruption [10].

Omalizumab is a humanized anti-immunoglobulin E antibody indicated for CIU/CSU in adults and adolescents who remain symptomatic despite H_1_-antihistamine treatment [13], as reported in the pivotal clinical studies [14–16]. Omalizumab, 150 or 300 mg, given as an every-4-week subcutaneous injection, significantly reduces disease activity, improves CIU/CSU symptoms, and is well tolerated in patients with refractory CIU/CSU symptoms. The 2013 international guidelines recommend the addition of omalizumab (150–300 mg) to current treatment for CIU/CSU patients who remain symptomatic despite receiving high doses of H_1_-antihistamines [17]. Phase III omalizumab CIU/CSU clinical studies collected sleep quality data via patient-reported outcome measures. We investigated the following research question: to what extent do sleep outcomes improve in CIU/CSU patients treated with omalizumab during 3 to 6 months within these three pivotal studies?

## Methods

### Study design

The omalizumab pivotal studies included ASTERIA I, ASTERIA II, and GLACIAL. Full study details are summarized elsewhere [14–16]. Briefly, each study included patients aged 12–75 years (18–75 years in Germany per regulatory requirements) with CIU/CSU who remained symptomatic despite treatment with background therapy. ASTERIA I and II allowed up to approved doses of H_1_-antihistamine [14, 16], while GLACIAL included patients being treated with up to 4 times approved doses plus H_2_-antihistamines, leukotriene receptor antagonists, or both [15]. In ASTERIA I and II, patients were randomized 1:1:1:1 to receive omalizumab 75, 150, 300 mg, or placebo subcutaneously every 4 weeks. Patients included in GLACIAL were randomized 3:1 to receive omalizumab 300 mg or placebo every 4 weeks. Dosing continued for 24 weeks in ASTERIA I and GLACIAL and for 12 weeks in ASTERIA II. Diphenhydramine 25 mg (up to 3 doses in 24 h) was allowed as a rescue medication for itch relief in all three studies.

In all three studies, patients were followed for an additional 16 weeks of observation after the end of the treatment period. Studies were conducted in accordance with US Food and Drug Administration regulations, the International Conference on Harmonisation E6 Good Clinical Practice guidelines, the principles of the Declaration of Helsinki, and any other applicable country laws. Study protocols were reviewed and approved by each institutional review board and all patients provided informed consent before study entry. The studies were registered with ClinicalTrials.gov, identifiers NCT01287117 (ASTERIA I), NCT01292473 (ASTERIA II), and NCT01264939 (GLACIAL).

### Patients

Patients were eligible for study inclusion if they met individual study age and background therapy requirements. Patients had a diagnosis of CIU/CSU for at least 6 months and presence of itch and hives for at least 8 (ASTERIA I and II) or more than 6 (GLACIAL) consecutive weeks before enrollment despite concurrent CIU/CSU therapy.

Patients reported their CIU/CSU symptoms and their impact on daily activities and sleep via an electronic daily diary [18] and paper-based patient-reported outcomes during office visits [baseline, weeks 4, 12, and 28 (ASTERIA II), and weeks 4, 12, 24, and 40 (ASTERIA II and GLACIAL)]. Individual study publications provide additional details on the study design [14–16].

### Sleep assessment

Sleep quality was assessed in all three studies via one question in the electronic diary [18], several questions in the Medical Outcomes Study Sleep Scale (MOS-Sleep Scale) [19], and the Chronic Urticaria Quality of Life Questionnaire (CU-Q_2_oL) [20].

In the Urticaria Patient Daily Diary (UPDD), a Sleep Interference question prompts patients to report the extent to which itch or hives interfered with their sleep in the previous 24 h (score range 0–3: 0 = no interference; 1 = mild, little interference with sleep; 2 = moderate interference, awoke occasionally, some interference with sleep; 3 = substantial, awake often, severe interference with sleep) [18]. A Weekly Sleep Interference Score (range 0–21) is the sum of the daily scores; a higher score indicates greater sleep interference. The UPDD has been validated in adults and adolescents with CIU/CSU [18, 21].

The MOS-Sleep Scale is a 12-item measure of sleep quality within a 4-week recall period [19]. The MOS-Sleep Scale has been validated in other disease areas, including diabetic neuropathic pain [22]. An MOS-Sleep scale score of 25.8 has been noted to be the population norm [23]. The MOS-Sleep Scale, Sleep Problem Index II (SPI-II) includes nine questions assessing sleep disturbance, sleep adequacy, somnolence, snoring, and awakening with shortness of breath or headache. SPI-II scores range from 0 to 100, with a higher number reflecting greater difficulty with sleep.

The CU-Q_2_oL is a 23-item questionnaire to measure quality of life in patients with chronic urticaria with a 2-week recall period [20]. The Sleep Problems dimension contains five questions about the extent to which urticaria has affected limited sleep, difficulty falling asleep, waking up during the night, tiredness during the day due to lack of sleep, difficulties with concentration, and feeling nervous. Each of item is scored on a five-point scale ranging from 1 (not at all) to 5 (extremely); thus the total scoring for this dimension ranges from 5 (least difficulty with sleep) to 25 (most difficulty with sleep).

### Statistical analysis

We compared the changes in MOS-Sleep Scale, UPDD Weekly Sleep Interference Score, and CU-Q_2_oL Sleep Problems domain scores from Baseline to each measurement point in the treatment arms with those in the placebo arms using analysis of covariance *t* tests, which were adjusted for baseline score (less than median, greater than or equal to median) and weight (<80, ≥80 kg) and individually fit at each time point. All statistical analyses were performed with SAS software (version 9.1; SAS Institute Inc., Cary, NC, USA).

We used Pearson correlation coefficient to estimate simple correlations among sleep outcomes in all three studies (pooled). We also explored the correlations between the trajectories of change in the Weekly Urticaria Activity Score (UAS7) and two domains of the MOS Sleep Scale (daytime somnolence and sleep disturbance) in the three studies. For that, we used latent growth modeling [24], wherein individual slopes of change and intercepts for UAS7 and MOS daytime somnolence and sleep disturbance were correlated for each patient.

To adjust for the use of diphenhydramine (as a rescue medication) and to properly model the correlated nature of within-patient observations over time, we conducted sensitivity analyses for the UPDD results using repeated-measures models, which adjusted for baseline score, weight, and weekly diphenhydramine dose as a time-varying covariate. A range of covariance structures was considered in fitting these repeated-measures models, all of which yielded very similar results. The final results presented are extracted from the models that used Toeplitz covariance structures, which assume that observations equally spaced in time have the same covariance.

## Results

Baseline patient characteristics have previously been reported [14–16]. At Baseline, patients with CIU/CSU reported substantial sleep impairment, with mean MOS-Sleep Scale, SPI-II scores ranging from 47.4 to 49.2, mean UPDD Weekly Sleep Interference Scores ranging from 11.2 to 12.6, and mean CU-Q_2_oL Sleep Problems domain scores ranging from 45.1 to 49.4 (Table 1).

**Table 1:** Baseline sleep impairment

Scale	ASTERIA I				ASTERIA II				GLACIAL	
	Placebo	Omalizumab 75 mg	Omalizumab 150 mg	Omalizumab 300 mg	Placebo	Omalizumab 75 mg	Omalizumab 150 mg	Omalizumab 300 mg	Placebo	Omalizumab 300 mg
MOS-Sleep Scale, SPI-II, n	80	77	80	81	79	82	82	79	83	252
Mean (SD)^a^	47.8 (19.8)	48.4 (17.8)	49.2 (21.2)	47.4 (19.3)	47.4 (18.1)	48.0 (18.6)	48.5 (18.6)	47.8 (17.8)	47.9 (18.6)	49.1 (18.9)
UPDD Weekly Sleep Interference Score, n	80	77	80	81	79	82	82	79	83	252
Mean (SD)	12.6 (4.8)	12.2 (5.3)	12.1 (5.2)	12.2 (4.5)	12.1 (4.5)	11.8 (5.4)	11.4 (5.6)	11.6 (4.3)	11.2 (5.2)	11.9 (4.8)
CU-Q_2_oL Sleep Problems domain, n	80	77	80	81	79	82	82	79	83	252
Mean (SD)^b^	48.7 (23.6)	46.8 (22.7)	48.2 (25.2)	49.3 (22.9)	46.6 (19.9)	45.1 (22.6)	46.4 (23.7)	49.4 (21.1)	46.5 (21.8)	46.4 (21.5)

*CU*-*Q*_*2*_ *oL* Chronic Urticaria Quality of Life Questionnaire, *MOS*-*Sleep Scale* Medical Outcomes Study Sleep Scale, *SPI*-*II* Sleep Problem Index II, *UPDD* Urticaria Patient Daily Diary

^a^Number of patients: ASTERIA I: omalizumab 75 mg, n = 76; ASTERIA II: placebo, n = 78; GLACIAL: placebo, n = 82; omalizumab 300 mg, n = 250

^b^Number of patients: ASTERIA I: placebo, n = 63; omalizumab 75 mg, n = 59; omalizumab 150 mg, n = 63; omalizumab 300 mg, n = 61; ASTERIA II: placebo, n = 69; omalizumab 75 mg, n = 70; omalizumab 150 mg, n = 70; omalizumab 300 mg, n = 71; GLACIAL: placebo, n = 79; omalizumab 300 mg, n = 243

On average, patients with CIU/CSU in all treatment arms experienced improvement in sleep at week 12 compared with Baseline for all sleep measures (Table 2). Patients treated with omalizumab reported a larger reduction in sleep problems than those in the placebo arm, and omalizumab 300 mg demonstrated best results among all study arms as noted in Table 2 [statistically significant improvement with omalizumab 300 mg at week 12 for MOS-Sleep Scale (all three studies), UPDD Weekly Sleep Interference Score (ASTERIA I and GLACIAL), and CU-Q_2_oL Sleep Problems domain (GLACIAL)]. Patient sleep improved quickly; the largest reduction in sleep problems was reported within the first 4 weeks of therapy and were maintained over the active treatment period. The patterns of sleep improvement are presented below according to each medium of assessment.

**Table 2:** Change from Baseline in sleep measures at week 12

Scale	ASTERIA I				ASTERIA II				GLACIAL	
	Placebo	Omalizumab 75 mg	Omalizumab 150 mg	Omalizumab 300 mg	Placebo	Omalizumab 75 mg	Omalizumab 150 mg	Omalizumab 300 mg	Placebo	Omalizumab 300 mg
MOS-Sleep Scale, SPI-II, n	63	67	63	72	70	70	70	73	62	217
Mean (SD)	−1.7 (18.0)	−14.4 (18.6)	−11.8 (20.6)	−18.1 (21.6)*	−10.8 (16.7)	−15.5 (20.0)	−16.4 (15.4)*	−16.8 (19.4)*	−13.4 (15.8)	−19.0 (20.5)***
UPDD Weekly Sleep Interference Score, n	62	64	63	73	69	68	73	74	67	214
Mean (SD)	−5.0 (5.3)	−7.0 (5.6)	−7.1 (6.0)*	−9.6 (5.3)**	−6.2 (5.7)	−6.8 (6.7)	−7.8 (6.4)	−9.2 (5.8)**	−5.0 (6.5)	−9.2 (5.3)**
CU-Q_2_oL Sleep Problems domain, n	49	50	48	57	62	59	61	65	60	210
Mean (SD)	−18.8 (23.5)	−20.5 (21.6)	−22.1 (25.4)	−30.2 (23.8)	−18.0 (24.2)	−22.6 (23.4)	−25.2 (26.4)	−33.3 (24.9)**	−18.3 (22.2)	−29.4 (23.8)**

*CU*-*Q*_*2*_ *oL* Chronic Urticaria Quality of Life Questionnaire, *MOS*-*Sleep Scale* Medical Outcomes Study Sleep Scale, *SPI*-*II* Sleep Problem Index II, *UPDD* Urticaria Patient Daily Diary

Adjusted * p < 0.05; ** p < 0.001; *** p < 0.01, versus placebo

### UPDD Weekly Sleep Interference Scores

**Fig. 1:**
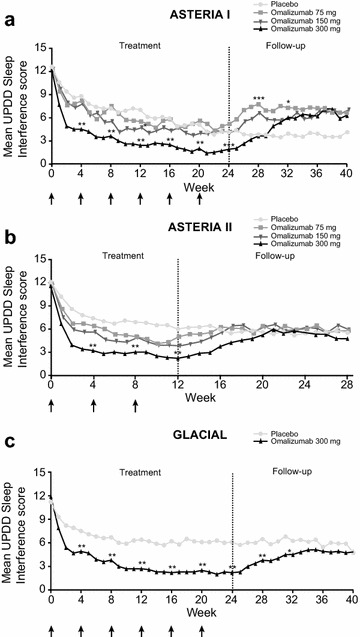
Change in UPDD Weekly Sleep Interference Score. **a** ASTERIA I, **b** ASTERIA II, and **c** GLACIAL. *Arrows* represent omalizumab dosing. *Low numbers* represent good quality sleep. Least-squares means were derived from a repeated-measures model adjusted for baseline value (less than median, greater than or equal to median) and baseline weight (<80, ≥80 kg). Statistical significance is marked every 4 weeks to minimize the visual burden of the graph. However, the following endpoints demonstrated statistical significance, in addition to the ones marked on the graph: ASTERIA I: all time points for omalizumab 300 mg during treatment and follow-up weeks 25, 34, 37 and 38; weeks 9, 10, 11, and 13 for omalizumab 150 mg; and weeks 26, 27, 29, 30, 31, 33, 34, 37 for omalizumab 75 mg; ASTERIA II: all time points for omalizumab 300 mg during treatment and follow-up weeks 13–15; weeks 1, 2, 3, 5, 6, 7, 9, 10, 11, 13, and 21 for omalizumab 150 mg; and weeks 1, 2, 10, 13, and 21 for omalizumab 75 mg; GLACIAL: all time points during treatment and follow-up weeks 25, 26, 27, 29, 30, and 31 for omalizumab 300 mg. *p < 0.05; **p < 0.001; ***p < 0.01, versus placebo. *UPDD* Urticaria Patient Daily Diary

As assessed through the UPDD Weekly Sleep Interference Score, patients reported improvement in sleep as early as week 1, and the most dramatic improvement was observed by week 4 (Fig. 1). Further sleep improvement was reported by week 12, and beyond, by week 24 (in ASTERIA I and GLACIAL). During active treatment in all three studies, the omalizumab 300 mg arm demonstrated the largest improvement compared with all other treatment arms as noted in Fig. 1 (statistical significance with omalizumab 300 mg at all time points during active treatment in all three studies). After the active treatment period during which improvements had been observed, all omalizumab-treated patients experienced a relapse in symptoms, including sleep, although the scores did not fully return to baseline values. Placebo-treated patients who had not experienced a substantial improvement in sleep measures during the active treatment period, continued with stable symptoms during the follow-up period.

Sensitivity analyses demonstrated similar trends in the data; sleep systematically improved over the course of the study, and the omalizumab 300 mg arm demonstrated the best outcomes (Additional file 1: Figure S1).

### MOS-Sleep Scale, SPI-II scores

**Fig. 2:**
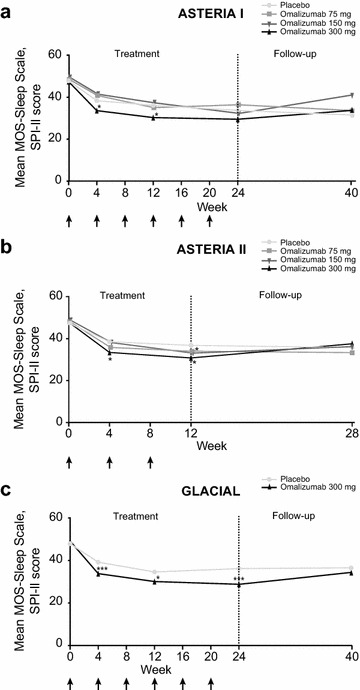
Change in MOS-Sleep Scale, SPI-II score: **a** ASTERIA I, **b** ASTERIA II, and **c** GLACIAL. *Arrows* represent omalizumab dosing. *Low numbers* represent good quality sleep. Least-squares means were derived from a repeated-measures model adjusted for baseline value (less than median, greater than or equal to median) and baseline weight (<80, ≥80 kg). *p < 0.05; **p < 0.001; ***p < 0.01, versus placebo. *MOS*-*Sleep Scale* Medical Outcomes Study Sleep Scale, *SPI*-*II* Sleep Problem Index II

**Fig. 3:**
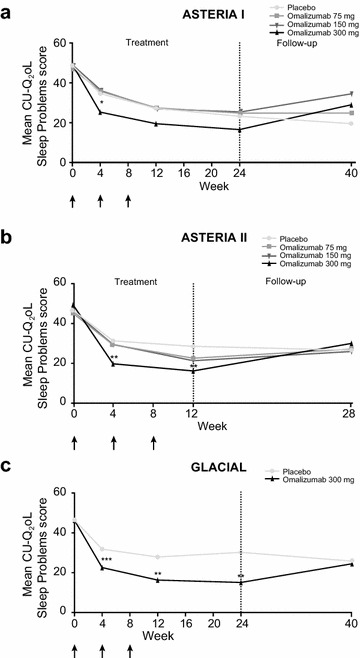
Change in CU-Q_2_oL Sleep Problems domain. **a** ASTERIA I, **b** ASTERIA II, and **c** GLACIAL. *Arrows* represent omalizumab dosing. *Low numbers* represent good quality sleep. Least-squares means were derived from a repeated-measures model adjusted for baseline value (less than median, greater than or equal to median) and baseline weight (<80, ≥80 kg). *p < 0.05; **p < 0.001; ***p < 0.01, versus placebo. *CU*-*Q*_*2*_ *oL* Chronic Urticaria Quality of Life Questionnaire

MOS-Sleep Scale, SPI-II scores reflected improvement in sleep at the first time of measurement, week 4, after which the improvement in sleep was maintained until weeks 12 (all studies) and 24 (ASTERIA I and GLACIAL; Fig. 2). In all studies, omalizumab 300 mg demonstrated the largest improvement compared with the other treatment arms as noted in Fig. 2 (statistically significant improvement with omalizumab 300 mg at all measured time points during active treatment in all three studies) (Fig. 3).

### CU-Q_2_oL scores

Through the CU-Q_2_oL questionnaire Sleep Problems domain, patients reported the most dramatic improvement in sleep at the first time of assessment (week 4). Afterwards, the improvement in sleep was maintained or slightly improved through weeks 12 (all studies) and 24 (ASTERIA I and GLACIAL; Fig. 3). In all three studies, sleep improvement was greatest with omalizumab 300 mg as noted in Fig. 5 (statistically significant improvement with omalizumab 300 mg at all measured time points during active treatment in ASTERIA II and GLACIAL and at weeks 4 and 8 in ASTERIA I).

### Correlation analyses

**Fig. 4:**
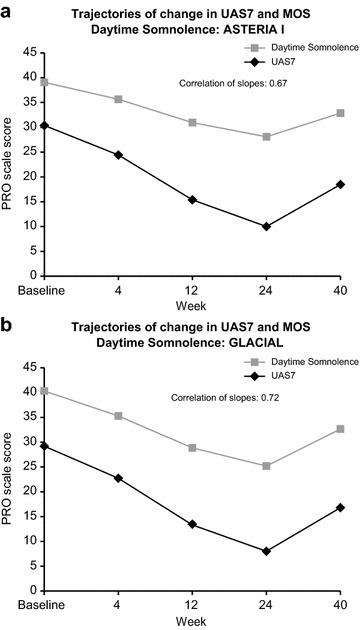
Correlations between change in UAS7 and change in MOS daytime somnolence: **a** ASTERIA I, **b** GLACIAL. *UAS7* Weekly Urticaria Activity Score, *MOS*-*Sleep Scale* Medical Outcomes Study Sleep Scale, *PRO* patient-reported outcomes

**Fig. 5:**
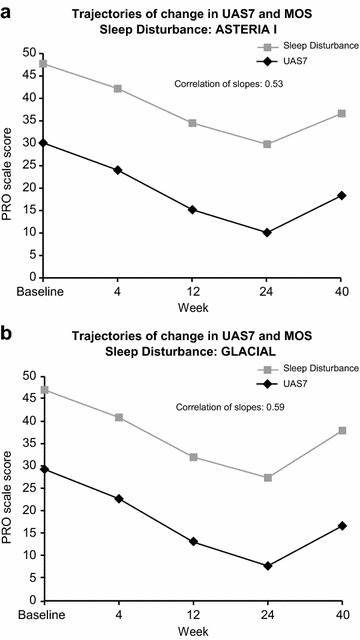
Correlations between change in UAS7 and change in MOS sleep disturbance: **a** ASTERIA I, **b** GLACIAL. *UAS7* Weekly Urticaria Activity Score, *MOS*-*Sleep Scale* Medical Outcomes Study Sleep Scale, *PRO* patient-reported outcomes

The correlations between sleep outcomes (Table 3) ranged from moderate to strong. The strongest correlation at all time-points was between CU-Q2oL and MOS Sleep Scale, Sleep Problem Index-II, possibly reflecting longer recall period (2 and 4 weeks, respectively) and broader scope of questions than in UPDD Weekly Sleep Interference Score, obtained through UPDD, which was completed daily. Latent curve modeling revealed correlation between changes in the disease activity and changes in sleep. Individual-level changes in UAS7 and MOS daytime somnolence were moderately to strongly correlated in ASTERIA I and GLACIAL (Fig. 4). Individual-level changes in the UAS7 and MOS sleep disturbance were moderately correlated in ASTERIA I and GLACIAL (Fig. 5). In other words, improvement in itch and hives was associated with decreases in somnolence and sleep disturbance.

**Table 3:** Pearson correlation analysis among sleep scores (pooled data)

	MOS-Sleep Scale, SPI-I	UPDD Sleep Interference Score	CU-Q_2_oL Sleep Problems domain
Sleep outcome			
Baseline			
MOS-Sleep Scale, SPI-I			
r	1.00000	0.39454*	0.74655*
n	970	970	847
UPDD Weekly Sleep Interference Score			
r	0.39454*	1.00000	0.48848*
n	970	975	848
CU-Q_2_oL Sleep Problems domain			
r	0.74655*	0.48848*	1.00000
n	847	848	848
Week 12			
MOS-Sleep Scale, SPI-I			
r	1.00000	0.44790*	0.72307*
n	828	809	722
UPDD Weekly Sleep Interference Score			
r	0.44790*	1.00000	0.60849*
n	809	827	707
CU-Q_2_oL Sleep Problems domain			
r	0.72307*	0.60849*	1.00000
n	722	707	724
Week 24			
MOS-Sleep Scale, SPI-I			
r	1.00000	0.462952*	0.68932*
n	494	475	435
UPDD Weekly Sleep Interference Score			
r	0.46295*	1.00000	0.58334*
n	475	726	418
CU-Q_2_oL Sleep Problems domain			
r	0.68932*	0.58334*	1.00000
n	435	418	437

*CU*-*Q*_*2*_ *oL* Chronic Urticaria Quality of Life Questionnaire, *MOS*-*Sleep Scale* Medical Outcomes Study Sleep Scale, *SPI*-*II* Sleep Problem Index II, *UPDD* Urticaria Patient Daily Diary

* p < 0.0001

## Discussion

Data from the three pivotal studies of omalizumab demonstrated substantial sleep improvement in patients with CIU/CSU. Marked improvement in sleep scores was observed after the first dose of therapy. As assessed by the three scales, the omalizumab 300 mg arms demonstrated the greatest improvements among all treatment arms. These results suggest that for patients with CIU/CSU and disturbed sleep, omalizumab should be considered as a potential treatment option, especially if the underlying CIU/CSU is uncontrolled despite H_1_-antihistamines.

Sleep disturbances in dermatologic disorders may substantially impair health-related quality of life and may be associated with serious psychopathology [25]. Sleep disorders in dermatologic conditions may result not only because of symptoms such as pruritus causing difficulties initiating or maintaining sleep (e.g., insomnia), but also through other physiologic mechanisms, including disruption of the skin’s thermoregulatory function [25, 26]. The treatment of sleep disturbance in dermatologic disorders such as atopic dermatitis has received considerable attention, but the impacts of treatment on sleep disturbance in CIU/CSU have not been well elucidated [27]. Moreover, treatments of sleep disturbance in dermatologic disorders often focus on response to sedating medications such as hypnotics rather than treatment of the underlying condition [27]. Therefore, it is notable that we observed improvements in sleep by targeting the underlying dermatologic disorder.

Grob et al. [8] reported on sleep impairment in 1356 adult patients with chronic skin disorders; three groups of patients were represented: chronic urticaria, psoriasis, and atopic dermatitis. Interference with sleep described as “often” or “every night” was reported by more than 50 % of patients with chronic urticaria. Sleeping was more problematic in patients with chronic urticaria and psoriasis than in those with atopic dermatitis. The baseline characteristics of our patient population with refractory CIU/CSU symptoms confirm that sleep impairment is problematic for these patients as reflected by measurement with the three patient-reported outcome measurement tools (MOS-Sleep Scale, SPI-II; UPDD Weekly Sleep Interference Score; and CU-Q_2_oL Sleep Problems domain).

Although sedating H_1_-antihistamines work as nighttime therapy for CIU/CSU (to relieve symptoms and help with sleep), some patients report their itch and hives persist despite such treatment. Over-the-counter diphenhydramine lacks consensus recommendation in the treatment of chronic insomnia, and some have questioned its efficacy and safety [28]. ASTERIA I and ASTERIA II demonstrated a dose-dependent reduction in the mean number of diphenhydramine tablets/week as compared with placebo at Week 12 (ASTERIA I: placebo, −1.00; 75 mg, −2.29, p = 0.1356; 150 mg, −2.94, p = 0.0249; 300 mg, −4.20, p = 0.0001; ASTERIA II: placebo, −2.21; 75 mg, −2.33, p = 0.9120; 150 mg, −3.72, p = 0.0682; 300 mg, −4.14, p = 0.0138); the reduction in GLACIAL was not significant (placebo, −2.70, 300 mg, −3.92, p = 0.1499). All enrollees in our clinical studies were patients with moderate to severe CIU/CSU who failed at least 6 weeks of therapy with licensed doses of H_1_-antihistamines. In GLACIAL, H_1_-antihistamines could be used at up to four times the approved dose, increasing the possibility of producing a sedating effect. This is consistent with previous reports that as much as 50 % of patients with CIU/CSU (especially with moderate to severe urticaria) fail to respond to up to four times the approved dose of H_1_-antihistamines [6, 29, 30].

Omalizumab use in these patients resulted in improved sleep, and this effect was persistent beyond the H_1_-antihistamine background therapy. In GLACIAL, patients were allowed to take an H_1_-antihistamine at up to four times the approved dose plus H_2_-antihistamines, leukotriene receptor antagonists, or both. In ASTERIA I, patients were allowed to add 1 additional H_1_-antihistamine at approved dosing after week 12. Omalizumab 300 mg demonstrated the best results throughout the duration of the study, with the omalizumab 300 mg arms exhibiting notable improvement in sleep and retaining it through the end of the active treatment period.

Sleep improvement recorded via UPDD Weekly Sleep Interference scores appeared to be more pronounced than those recorded via CU-Q2oL or MOS-SS. Moreover, the scores of the latter two instruments correlated stronger between themselves than they did with the WSI scores. This may appear counter-intuitive in light of the fact that MOS-SS is a sleep-specific instrument. The root of this phenomenon may lay in the intricacies of the disease and instruments’ ability to detect change. Because CIU/CSU symptoms can wax and wane throughout a course of days and even hours, daily symptoms capture offered by the eDiary (the tool used to collect data from WSI scores) rendered more accurate picture of the drug effect on the disease than the CU-Q2oL and MOS-SS. Because their recall periods comprise 2 and 4 weeks, respectively, CU-Q2oL and MOS-SS instruments tend to capture the “overall sleep” picture as opposed to the volatility of sleep directly related to CIU/CSU.

Results should be interpreted in light of limitations. Minimally important clinical differences for changes in sleep measures have not been established in patients with CIU/CSU. These analyses are limited by the treatment period of the clinical studies; response to treatment for extended periods is not known. Also, the use of over-the-counter sleep aids was not measured in our study. Additionally, although the improvements in patients’ underlying CIU/CSU would seem to suggest that this is the etiology of improved sleep, we cannot determine the extent to which this may have been because of reduced pruritus versus other potential etiologies, such as a more direct physiologic effect of improved dermatologic health. However, improvements in symptoms of CIU/CSU associated with omalizumab treatment in the Phase III trials have been shown to correlate closely with improvements in health-related quality of life [31].

## Conclusions

Patients with CIU/CSU commonly reported sleep impairment at Baseline. Patients treated with omalizumab reported improvement in sleep after the first dose of omalizumab. Sleep continued to improve with subsequent doses, and sleep improvement was evident until the end of the active treatment period. The return of disease symptoms following discontinuation of active treatment was accompanied by a resurgence of sleep impairment. Patients receiving omalizumab 300 mg achieved greater improvement in sleep than those in other treatment arms.

## Additional file

10.1186/s13601-016-0120-0 Sensitivity analysis: changes in UPDD Sleep Interference score: **a** ASTERIA I, **b** ASTERIA II, and **c** GLACIAL. Arrows represent omalizumab dosing. Lower numbers represent better sleep. Least-squares means were derived from a repeated-measures model adjusted for baseline value (<median, ≥median), baseline weight (<80 kg, ≥80 kg), and weekly rescue medication (diphenhydramine) dose. Statistical significance is marked every 4 weeks to minimize the visual burden of the graph. However, the following endpoints demonstrated statistical significance, in addition to the ones marked on the graph: ASTERIA I: all time points for omalizumab 300 mg; weeks 2, 3, 7, 9, 10, 11, and 13 for omalizumab 150 mg; and weeks 1, 2, 5, 6, 10, 11, and 13 for omalizumab 75 mg; ASTERIA II: all time points for omalizumab 300 mg; weeks 1, 2, 3, 5, 6, 7, 9, 10, and 11 for omalizumab 150 mg; and weeks 1, 2, 5, 7, 9, 10, and 11 for omalizumab 75 mg; GLACIAL: all time points for omalizumab 300 mg. *p < 0.05; **p < 0.001; ***p < 0.01, versus placebo. UPDD: Urticaria Patient Daily Diary.

## Abbreviations

CIU: chronic idiopathic urticaria
CSU: chronic spontaneous urticaria
CU-Q_2_oL: Chronic Urticaria Quality of Life Questionnaire
MOS-Sleep Scale: Medical Outcomes Study Sleep Scale
SPI-II: Sleep Problem Index II
UPDD: Urticaria Patient Daily Diary
