# Supplementary figures and images for: Improvement of sleep in patients with chronic idiopathic/spontaneous urticaria treated with omalizumab: results of three randomized, double-blind, placebo-controlled studies
Source: Clin Transl Allergy. 2016 Aug 18;6:32. doi: 10.1186/s13601-016-0120-0 (PMC4989527; doi:10.1186/s13601-016-0120-0)

**a**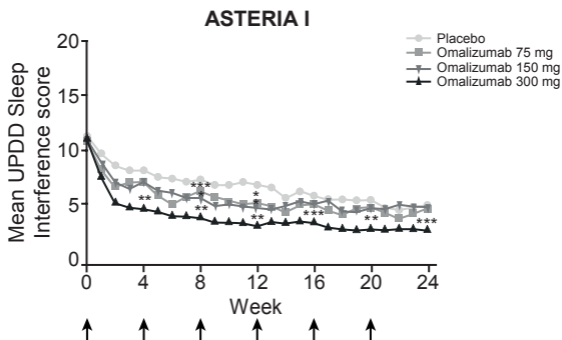**b**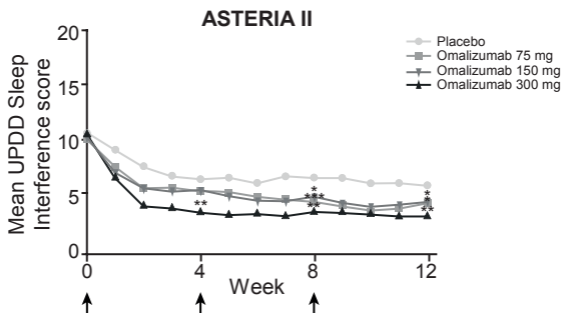**c**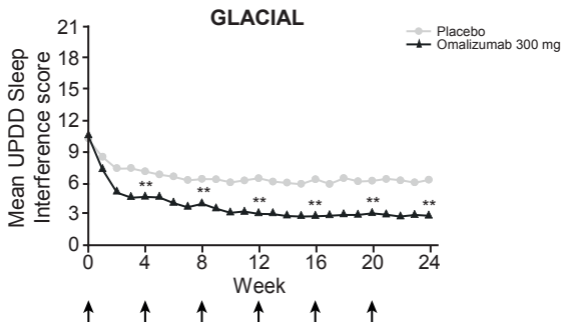

Supplement: Supplementary file 1 — 10.1186/s13601-016-0120-0 Sensitivity analysis: changes in UPDD Sleep Interference score: a ASTERIA I, b ASTERIA II, and c GLACIAL. Arrows represent omalizumab dosing. Lower numbers represent better sleep. Least-squares means were derived from a repeated-measures model adjusted for baseline value (<median, ≥median), baseline weight (<80 kg, ≥80 kg), and weekly rescue medication (diphenhydramine) dose. Statistical significance is marked every 4 weeks to minimize the visual burden of the graph. However, the following endpoints demonstrated statistical significance, in addition to the ones marked on the graph: ASTERIA I: all time points for omalizumab 300 mg; weeks 2, 3, 7, 9, 10, 11, and 13 for omalizumab 150 mg; and weeks 1, 2, 5, 6, 10, 11, and 13 for omalizumab 75 mg; ASTERIA II: all time points for omalizumab 300 mg; weeks 1, 2, 3, 5, 6, 7, 9, 10, and 11 for omalizumab 150 mg; and weeks 1, 2, 5, 7, 9, 10, and 11 for omalizumab 75 mg; GLACIAL: all time points for omalizumab 300 mg. *p < 0.05; **p < 0.001; ***p < 0.01, versus placebo. UPDD: Urticaria Patient Daily Diary. [file 13601_2016_120_MOESM1_ESM.pdf]
